# Retraction

**DOI:** 10.4084/MJHID.2014.024

**Published:** 2014-03-22

The article deposited in PUBMED as "Saad K, Mohamed SA, Metwalley KA. Recurrent/Persistent Pneumonia among Children in Upper Egypt. Mediterr J Hematol Infect Dis. 2013 Apr 18;5(1):e2013028. doi: 10.4084/MJHID.2013.028. Print 2013. PubMed PMID: 23667726;PubMed Central PMCID: PMC3647710." has been removed from Mediterr J Hematol Infect Dis because the paper was previously published in another journal.

